# Langerin-expressing dendritic cells in human tissues are related to CD1c^+^ dendritic cells and distinct from Langerhans cells and CD141^high^ XCR1^+^ dendritic cells

**DOI:** 10.1189/jlb.1HI0714-351R

**Published:** 2014-12-16

**Authors:** Venetia Bigley, Naomi McGovern, Paul Milne, Rachel Dickinson, Sarah Pagan, Sharon Cookson, Muzlifah Haniffa, Matthew Collin

**Affiliations:** Human Dendritic Cell Laboratory, Institute of Cellular Medicine, Newcastle University, Newcastle upon Tyne, United Kingdom

**Keywords:** antigen presenting cell, differentiation, HSCT, DCML deficiency, GATA2

## Abstract

Langerin is a C-type lectin expressed at high level by LCs of the epidermis. Langerin is also expressed by CD8^+^/CD103^+^ XCR1^+^ cross-presenting DCs of mice but is not found on the homologous human CD141^high^ XCR1^+^ myeloid DC. Here, we show that langerin is expressed at a low level on DCs isolated from dermis, lung, liver, and lymphoid tissue and that langerin^+^ DCs are closely related to CD1c^+^ myeloid DCs. They are distinguishable from LCs by the level of expression of CD1a, EpCAM, CD11b, CD11c, CD13, and CD33 and are found in tissues and tissue-draining LNs devoid of LCs. They are unrelated to CD141^high^ XCR1^+^ myeloid DCs, lacking the characteristic expression profile of cross-presenting DCs, conserved between mammalian species. Stem cell transplantation and DC deficiency models confirm that dermal langerin^+^ DCs have an independent homeostasis to LCs. Langerin is not expressed by freshly isolated CD1c^+^ blood DCs but is rapidly induced on CD1c^+^ DCs by serum or TGF-*β* via an ALK-3-dependent pathway. These results show that langerin is expressed outside of the LC compartment of humans and highlight a species difference: langerin is expressed by the XCR1^+^ "DC1" population of mice but is restricted to the CD1c^+^ "DC2" population of humans (homologous to CD11b^+^ DCs in the mouse).

## Abstract (precis)

Langerin is not restricted to Langerhans cells, but expressed at low levels by CD1c^+^ dendritic cells and is inducible by TGFβ in humans.

## Introduction

Human skin and other nonlymphoid tissues contain several subsets of DCs and macrophages [1, 2]. In addition to LCs of stratified epidermis, the interstitial tissues contain CD1c^+^ myeloid DCs and a minor population of CD141^high^ myeloid DCs that mirror the CD1c^+^ and CD141^+^ blood DC populations [3]. CD141^+^ myeloid DCs are homologous to CD8^+^ and CD103^+^ DCs in mice that express XCR1 and have enhanced cross-presentation capacity. XCR1^+^ DCs have been referred to as "DC1" and the CD1c^+^ myeloid DC as "DC2" in a recently proposed classification of mammalian DCs [2].

Human LCs are characterized by high expression of langerin, a C-type lectin, and CD1a. Interstitial tissue DCs also express CD1a but at a lower level to that found on LCs [3–9]. Dermal and lung CD1a^+^/CD1c^+^ DCs (DC2) have been reported previously to be langerin negative [7, 10], although langerin expression was reported in the lamina propria of human colon [11].

The selective expression of pattern recognition receptors, including lectins and TLRs, confers specialized functions to different populations of DCs. Langerin is able to bind viruses and other pathogens, is implicated in antigen processing, and is found within the Birbeck granule of LCs [12–14]. These observations do not exclude a role for langerin in other DC populations.

In mice, langerin is also expressed by XCR1^+^ cross-presenting DCs (DC1), expressing CD103^+^ in the tissues or CD8^+^ in the lymphoid organs [15–20]. In humans, however, the DC1 subset of CD141^high^ XCR1^+^ myeloid DCs is langerin negative [3].

In human progenitor cells and monocytes, langerin is induced by a wide range of cytokines in vitro, including GM-CSF, TNF, TGF-*β*, and BMP-7 [8, 21–24]. These results suggest that langerin might be expressed by other DCs outside of the LC compartment. In this report, we set out to re-examine the question of langerin expression by human DCs in situ and found evidence of langerin expression by CD1c^+^ myeloid DCs.

## MATERIALS AND METHODS

### Samples and patient data

Blood, skin, and surplus biopsy material was obtained from healthy human volunteers and hematopoietic transplant patients under ethical approval from the Newcastle and North Tyneside Research Ethics Committee 1. Skin, tissues, and blood were processed as described previously [3].

### Flow cytometry and cell sorting

Cells were stained in aliquots of up to 2 × 10^6^ cells in 50 *μ*l buffer, according to standard protocols. Nonviable cells were excluded by staining with DAPI (Partec, Sysmex, Görlitz, Germany) or LIVE/DEAD dead cell stain (Invitrogen, Carlsbad, CA, USA). Flow cytometry was performed on a LSRII cytometer (Becton Dickinson, Franklin Lakes, NJ, USA) and data analyzed with FlowJo (Treestar, Ashland, OR, USA). FACS was performed by use of a BD FACSAria (100 *μ*m nozzle and 20 pounds/square inch; BD Biosciences, San Jose, CA, USA). Sorted cells were collected into Eppendorfs containing RPMI with 10% FCS or sorted directly onto glass slides, air dried, and fixed in methanol. Antibodies were from Becton Dickinson unless stated otherwise (clone in parentheses). CD1a-FITC (NA1/34; Dako, Glostrup, Denmark), CD1a-APC (HI 149), CD1c-PE/APC (AD5-8E7; MACS; Miltenyi Biotec, Bergisch Gladbach, Germany), CD3-FITC (UCHT1), CD11b-APC (ICRF44; BioLegend, San Diego, CA, USA), CD11c-APC/V450 (B-ly6), CD13-APC (WM15), CD14-FITC/PE/PE-Cy7 (M5E2), QDOT605 (TuK4; Invitrogen), CD16-FITC/PE-Cy7 (3G8), CD19-FITC (SJ25C1), CD20-FITC (L27), CD31-FITC (WM59), CD34-APC (8G12)/APC-Cy7 (581; BioLegend), CD45-APC-Cy7/V450 (2D1), CD56-FITC (NCAM16.2), CD115-PE (61708; R&D Systems, Minneapolis, MN, USA), CD123-PE/PerCP-Cy5.5 (9F5), CD135-PE (4G8), CD141-PE (1A4), CD141-APC (AD5-14H12; MACS; Miltenyi Biotec), HLA-DR-PerCP-Cy5.5 (L243), langerin-PE (DCGM4; Beckman Coulter, Brea, CA, USA), and EpCAM-APC (EBA-1).

### Microscopy and FISH

Fluorescence microscopy was performed by use of a Zeiss AxioPlan 2 microscope EC Plan-Neofluar ×40 numerical aperture 0.75 lens and AxioCam running Zeiss AxioVision v2.8 software (Zeiss, Thornwood, NY, USA). Separated epidermal and dermal sheets were fixed in acetone for 20 min and rehydrated in PBS for 20 min before staining. LCs were stained with CD1a-FITC (NA1/34; Dako), and langerin^+^ dermal DCs were visualized with HLA-DR FITC (L243; Becton Dickinson), polyclonal rabbit anti-langerin followed by Alexa Fluor 555-conjugated goat anti-rabbit IgG H+L (Invitrogen), and mouse monoclonal anti-CD11c (Becton Dickinson) followed by Alexa Fluor 647 (Invitrogen). Sorted skin DC subsets were spun onto glass slides, fixed in methanol, and restained for HLA-DR and langerin as above and mounted in Vectashield with DAPI (Vector Laboratories, Burlingame, CA, USA). For FISH, directly sorted skin cells were fixed in Carnoy’s (methanol: acetic acid 3:1). Vysis CEP X SpectrumOrange and CEP X SpectrumGreen DNA probe kit (Abbott Laboratories, Abbott Park, IL, USA) were used, according to the manufacturer’s instructions.

### Langerin Induction on blood DCs

Unfractionated PBMCs (2 × 10^6^) were incubated in 1 ml RPMI with 10% FCS for 18 h, with or without TGF-*β* (100 ng/ml). In experiments with sorted cells, 10,000 cells were cultured in 100 *μ*l. Cells were stained in the same Eppendorfs for surface expression of langerin and analyzed by flow cytometry initially and after 18 h. Recombinant human BMPR-IA/ALK-3 Fc chimera was obtained from R&D Systems (315-BR-100) and used at 2 *μ*g/ml.

### qPCR

RNA was extracted from sorted cell populations or LCH lesions by use of the Qiagen Micro kit (Qiagen, Valencia, CA, USA), according to the manufacturer’s instructions. Single-stranded cDNA was generated by use of an Moloney Murine Leukemai Virus Reverse Trnascriptase (M-MLV RT) kit (Invitrogen) with deoxyribonucleotide triphosphates (Roche, Indianapolis, IN, USA), random hexamers (Pharmacia, Uppsala, Sweden), and RNasin (Promega, Madison, WI, USA). qPCR reactions were performed with TaqMan Gene Expression Assays (Applied Biosciences, Life Technologies, Carlsbad, CA, USA) by use of the 7900HT Fast Real-Time PCR system (Applied Biosciences, Life Technologies). GAPDH was used to calculate ΔCT.

### Statistical analysis

Graphs were plotted with Prism Version 5.0a (GraphPad Software, La Jolla, CA, USA). Mean and sd calculation, paired *t*-tests, and linear-regression analysis were performed within the graphing software. All *P* values were two tailed. Heat maps of median fluorescence intensity were generated by use of MultiExperiment Viewer (http://www.tm4.org/index.html; TM4 Microarray Software Suite).

## RESULTS

### Langerin expression on a fraction of CD1a/c^+^ DCs in normal human tissues

**Figure 1.**
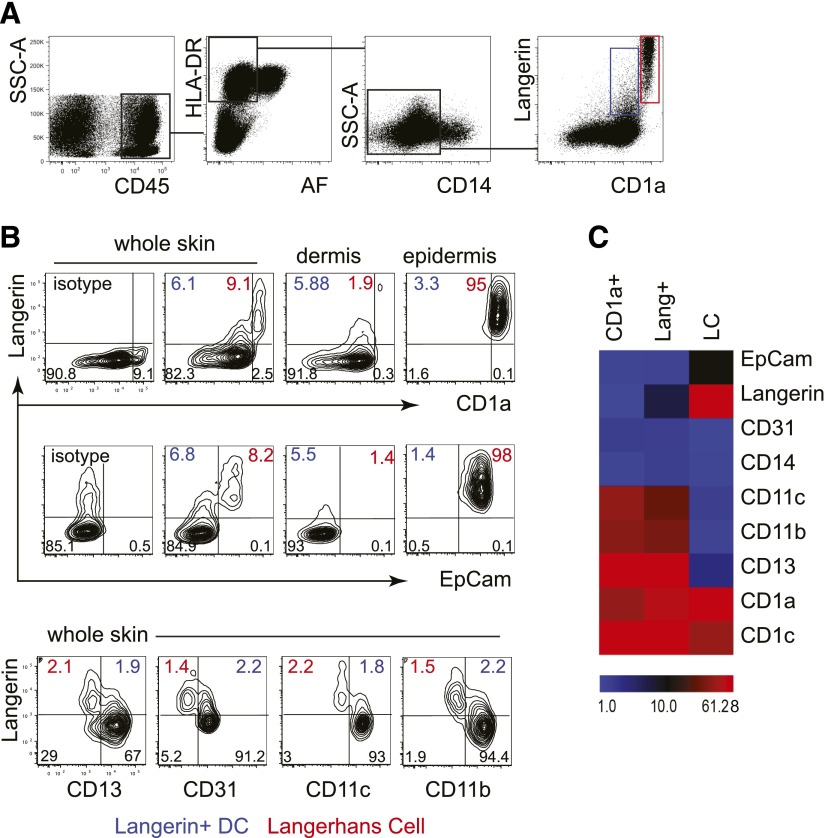
Langerin expression by cutaneous DCs, distinct from LCs. (A) Identification of langerin^+^ DCs among the CD45^+^, DR^+^, low autofluorescnce (AF) fraction of collagenase-digested skin. LCs are seen in the skin as CD1a^high^ langerin^high^ cells (red gate); a second CD1a^+^ langerin^+^ population is apparent (blue gate). Representative example of 22 experiments. SSC-A, Side-scatter-area. (B) Comparison of whole skin, dermis, and epidermis preparations, showing that langerin^+^ DCs are found in whole skin or dermis but not epidermis and may be separated from LCs by lower expression of CD1a and EpCAM or by higher expression of CD13, CD33, CD11c, and CD11b. Percentage of langerin^+^ DC (blue) and LCs are indicated (red). A representative example of 5 experiments shown. (C) Heat map summary of mean fluorescence intensity of key surface antigens of langerin^+^ DCs, CD1a^+^ dermal DCs, and LCs (mean of 4 experiments).

Collagenase-digested whole skin was analyzed by flow cytometry for langerin expression. From live CD45^+^ HLA-DR^+^ cells, fixed macrophages and monocyte-derived cells were excluded by gating out autofluorescent and CD14^+^ cells. Two populations of langerin^+^ cells were observed within the remaining population; one with high langerin and CD1a and the other with intermediate langerin and CD1a (**Fig. 1A**). Neither CD14^+^ cells nor the CD141^high^ subset of DCs expressed langerin, as described previously [3].

This observation suggested that in addition to langerin^high^ CD1a^high^ LCs originating from the epidermis, there was a lower level of langerin expression by CD1a^+^ dermal DCs. To define this further, dermis and epidermis were examined separately for langerin expression together with CD1a and EpCAM to distinguish LCs (CD1a^high^ EpCAM^+^) from dermal DCs (CD1a^low^ EpCAM^−^). This consistently demonstrated langerin^+^ dermal DCs (Fig. 1B). Additional antigens CD13, CD31, CD11c, and CD11b were able to dissociate LCs from langerin^+^ DCs (Fig. 1B). Comparison of all surface antigens analyzed suggested that langerin^+^ DCs were closely related to CD1a^+^ dermal DCs and distinct from LCs. All subsets expressed CD1c (Fig. 1C).

**Figure 2.**
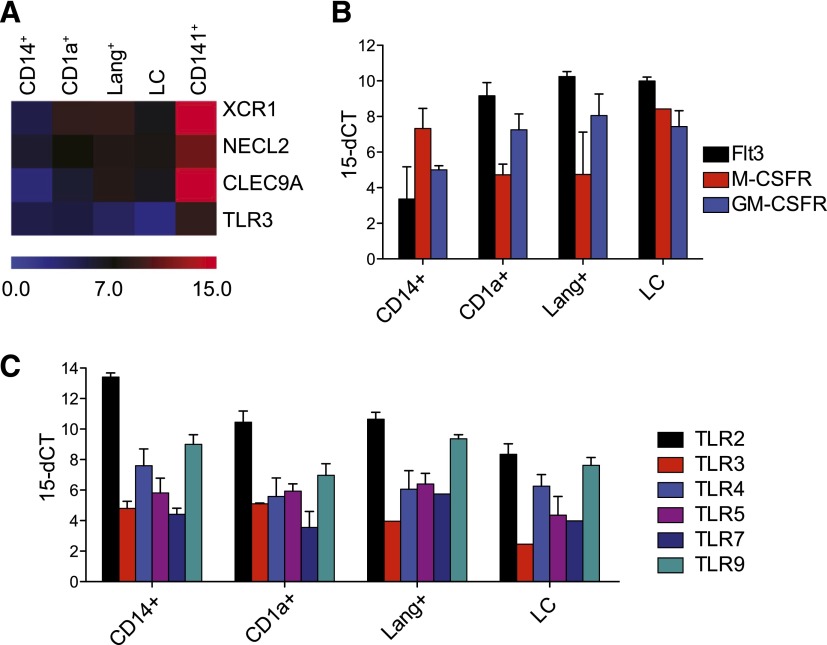
Gene-expression profiles of langerin^+^ DCs compared with other dermal DCs and LCs. (A) Heat map of qPCR for 4 characteristic transcripts of CD141^+^ cross-presenting DCs. Mean of 3 experiments. (B) Relative expression of growth factor receptors. Mean ± sem of 3 experiments. (C) Relative expression of TLRs. Mean + sem of 3 experiments

The phenotype of langerin^+^ DCs was explored further with qPCR. First, comparison with the signature of CD141^high^ XCR1^+^ DCs showed that dermal langerin^+^ DCs did not express the characteristic markers of cross-presenting DCs: XCR1, NECL2, and CLEC9 (**Fig. 2A**). Growth factor receptor expression profiles showed that langerin^+^ DCs had a high Flt-3/low M-CSFR signature in common with CD1a^+^ dermal DCs and distinct from LCs and CD14^+^ monocyte-derived cells (Fig. 2B). The TLR profile of langerin^+^ DCs was similar to that of CD1a^+^ dermal DCs, although all myeloid cells expressed a similar array of receptors (Fig. 2C).

**Figure 3.**
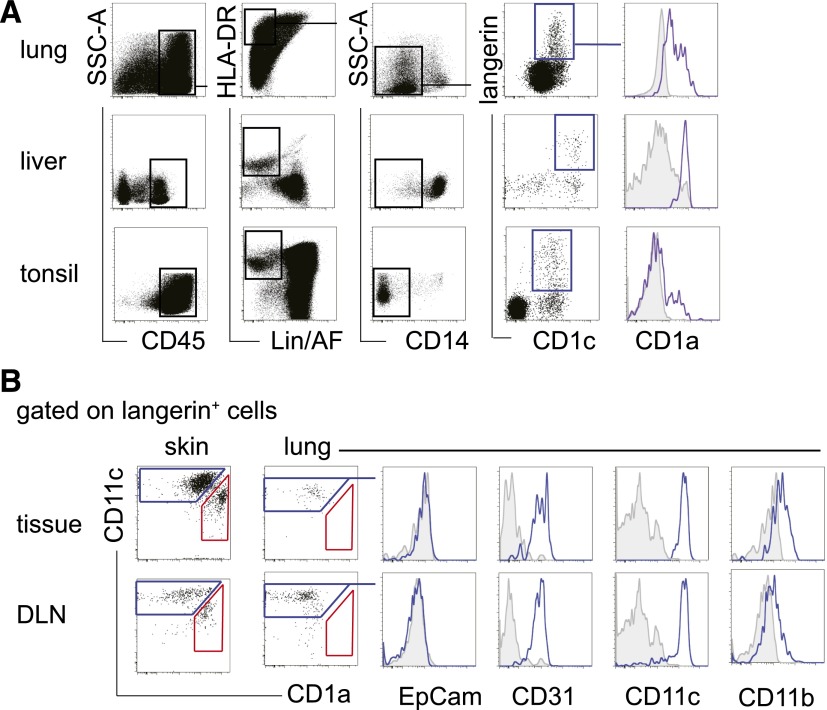
Langerin+ DCs in other tissues and draining LNs. (A) Langerin^+^ DCs identified within CD1c^+^ DCs of lung, liver, and tonsil, showing variable coexpression of CD1a. As in the dermis, langerin^+^ DCs were found in CD45^+^ HLA-DR^+^ lineage and autofluorescence negative (Lin^−^/AF^−^) CD14^−^ fraction. Data represent 3, 3, and 7 samples of lung, liver, and tonsil, respectively. (B) Detailed phenotype of langerin^+^ cells isolated from lung and lung draining LN (DLN). Skin is shown for comparison. Both skin and skin draining nodes contain langerin^+^ DCs (blue gate) and CD11c^low^ CD1a^high^ LCs (red gate). The langerin^+^ populations of lung and lung draining LNs contain only langerin^+^ DCs but no LCs. These both have a similar phenotype to dermal langerin^+^ DCs and are EpCAM^−^ CD11b^low^ CD31^+^ CD11c^+^.

Langerin^+^ CD1c^+^ DCs were also detectable in tissues normally devoid of LCs, including the lung, liver, and tonsil. There was variable expression of CD1a; higher levels were detected in the lung than liver or tonsil (**Fig. 3A**).

We examined skin and lung with matched draining LNs to determine whether the phenotype of langerin^+^ DCs in draining LNs was concordant with the tissues (Fig. 3B). Matched populations of langerin^+^ DCs and LCs, separable by CD11c and CD1a expression, were observed in skin and axillary LNs. In the lung and bronchial LNs, only langerin^+^ DCs but no LCs were found. The langerin^+^ DCs from both tissue and corresponding LN had a similar phenotype (Fig. 3B).

**Figure 4.**
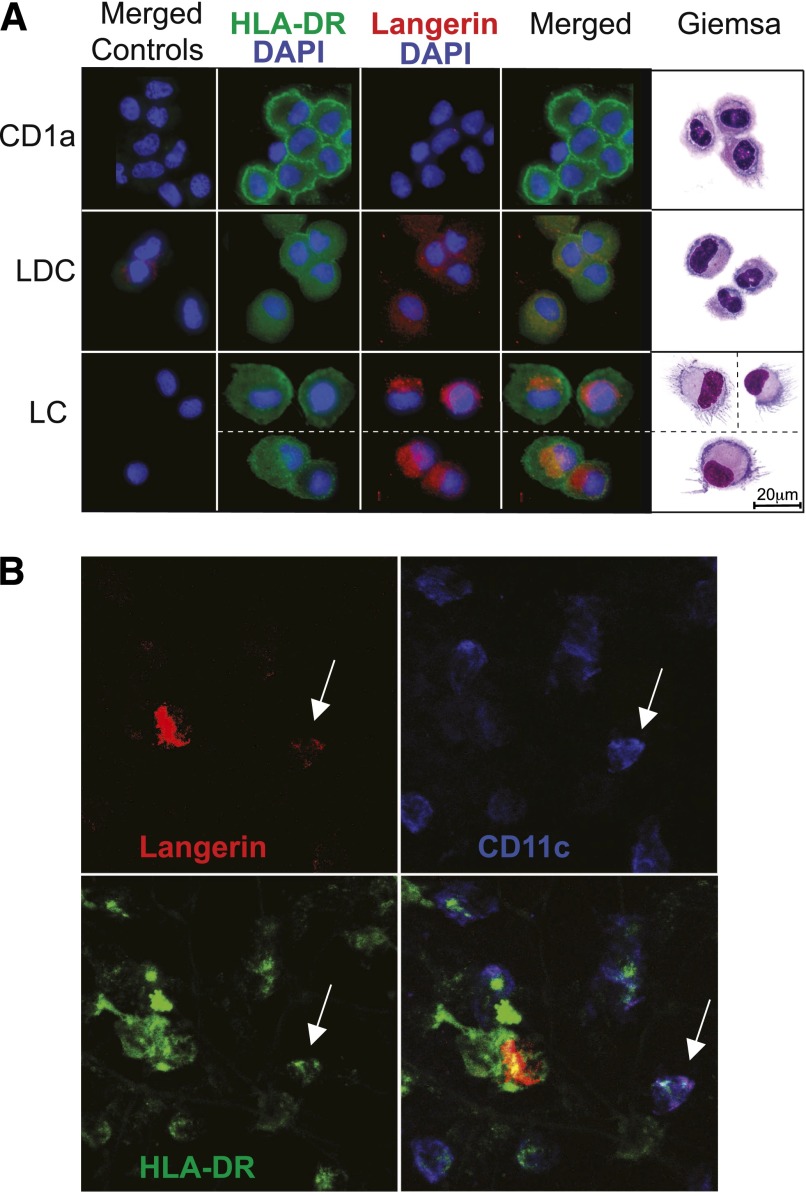
In situ expression of langerin by dermal DCs. (A) Sorted CD1a^+^ dermal DCs, langerin^+^ DCs (LDC) and LCs from freshly digested whole skin were spun onto cytopsin slides and reprobed with antibodies for HLA-DR and langerin. One of 2 independent experiments is shown. (B) In situ detection of langerin^+^ DCs in a whole-mount stain of dermal sheet (apical surface). The field contains a migratory LC with high langerin expression but no CD11c, a CD11c^+^ langerin^+^ DC (arrow) and a number of langerin^−^ CD11c^+^ myeloid DCs. Data taken from 1 of 5 experiments.

Immunofluorescence staining of sorted LCs, langerin^+^ CD1a^+^ DCs, and langerin^−^ CD1a^+^ DCs revealed diffuse cytoplasmic staining in langerin^+^ DCs, contrasting with the intense perinuclear Golgi staining of LCs. Langerin^+^ DCs were smaller than LCs and resembled CD1a^+^ DCs in appearance (**Fig. 4A**). With the use of CD11c expression to separate langerin^+^ DCs from LCs, it was possible to detect occasional langerin^+^ DCs in situ in the apical dermis. These also showed a similar diffuse pattern of langerin staining in contrast to the bright langerin expression of migrating LCs (Fig. 4B). Several attempts were made to detect Birbeck granules in sorted populations of langerin^+^ DCs, but none was found (not shown).

### The homeostasis of langerin^+^ dermal DCs is distinct from that of LCs

Maturing LCs down-regulate langerin expression and may acquire the characteristics of myeloid DCs. To verify that langerin^+^ dermal DCs were not derived from LCs, we examined 2 in vivo models of human DC homeostasis: HSCT and human DC deficiency as a result of *GATA2* mutation.

**Figure 5.**
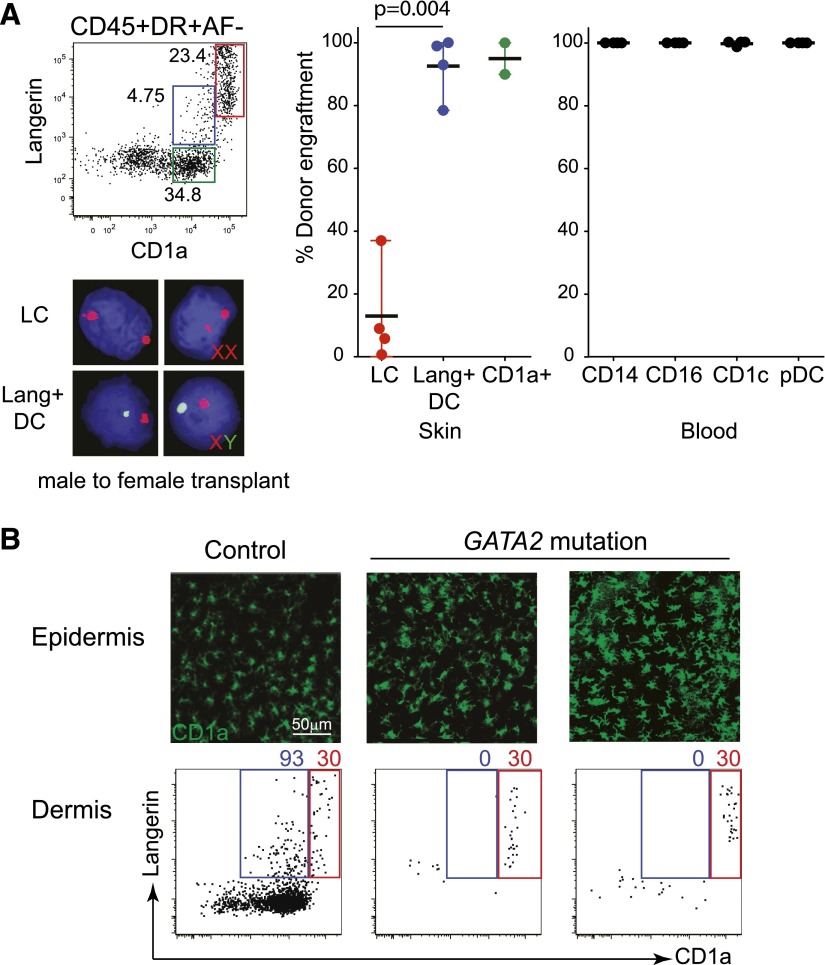
Homeostasis of langerin^+^ DCs compared with CD1a^+^ DCs and LCs. (A) Comparison of the level of donor engraftment at day 28 post-HSCT among dermal CD1a^+^ DCs (green gate and symbols), langerin^+^ DCs (blue gate and symbols), LCs (red gate and symbols), and peripheral blood monocytes and DCs (black). Cells were sorted as indicated on the dot plot and probed with X-Y FISH, as shown in the example. Four samples were analyzed. pDC, Plasmacytoid DC. (B) Analysis of LCs and dermal DCs in 2 patients with failure of bone marrow-derived DC hematopoiesis as a result of *GATA-2* mutation. A healthy control is shown on the left for comparison. (Upper) LC in situ in the epidermis; (lower) a small number of LCs crossing the dermis (red gates), in the absence of any detectable langerin^+^ or CD1a^+^ dermal DCs (blue gates).

In patients receiving HSCT, LCs remain predominantly recipient in origin for the first 28 d post-transplant, whereas donor-derived cells replace the blood and interstitial DC compartments [9, 25]. Langerin^+^ DCs and LCs were isolated from skin biopsies at day 28 of sex-mismatched transplants and probed with X-Y FISH. This indicated that langerin^+^ DCs were replaced rapidly by donor-derived cells (80–90%) in parallel with CD1a^+^ dermal DCs, blood monocytes, and blood DCs. All of these populations engrafted with much more rapid kinetics than LCs, which were 10–20% donor at the same time-point. These results indicate that langerin^+^ DCs are unlikely to be derived from maturing epidermal LCs crossing the dermis, as the latter were mostly recipient at the time of analysis (**Fig. 5A**).

Patients with DC deficiency as a result of *GATA2* mutations lack interstitial DC populations but maintain epidermal LCs [26, 27]. Closer analysis of these patients revealed that langerin^+^ DCs are also missing in parallel with CD1a^+^ dermal DCs. In contrast, the persistence of LCs, including the small afferent population crossing the dermis, further supports the conclusion that langerin^+^ dermal DCs are unlikely to be derived from LCs (Fig. 5B).

### Induction of langerin expression on CD1c^+^ blood myeloid DCs

**Figure 6.**
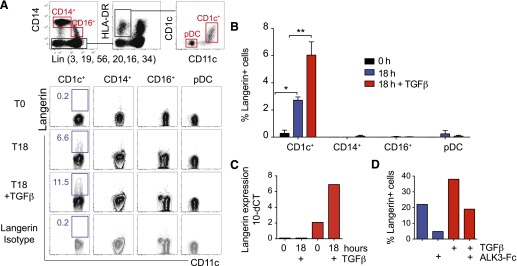
Induction of langerin in CD1c^+^ blood myeloid DCs. (A) Langerin induction on whole-cultured PBMC after 18 h (T18) of culture in vitro, with or without TGF-*β*, compared with isotype control. DCs and monocytes were identified by the gating strategy shown and the expression of langerin displayed below. Representative example of 4 experiments. (B) Langerin induction on sorted monocytes and blood DCs after 18 h of culture in serum containing medium and TGF-*β*. *P < 0.05; **P < 0.01. Mean of 4 experiments. (C) Expression of langerin mRNA by CD14^+^ monocytes (blue bars) and CD1c^+^ blood DCs (red bars) at 0 h and 18 h with TGF-*β*. One of 3 experiments is shown. (D) Inhibition of langerin induction in serum containing medium and TGF-*β* by an ALK-3-Fc chimeric-blocking agent. One of 3 experiments is shown.

Having observed langerin^+^ DCs in human tissues, peripheral blood was examined for langerin expression. None was detectable on freshly isolated cells, but langerin was induced at a low level in serum-containing medium and by TGF-*β* after 18 h of culture of unfractionated PBMC. Flow analysis after 18 h indicated that langerin was expressed selectively by CD1c^+^ myeloid DCs (**Fig. 6A**). Addition of TNF-*α* or GM-CSF, cytokines that are known to derive langerin^+^ DCs, did not further augment the expression of langerin, and langerin induction was not observed on the small number of CD141^+^ DCs present in these experiments (not shown). Sorted CD1c^+^ myeloid DCs, but not other populations, also expressed langerin after 18 h with serum-containing medium or with TGF-*β* (Fig. 6B). qPCR for langerin showed that freshly isolated CD1c^+^ DCs express a low level of langerin message that was up-regulated in culture with TGF-*β*. This is in contrast to CD14^+^ monocytes, which remain negative (Fig. 6C). It was possible to reduce the level of langerin induction by TGF-*β* with the use of an ALK-3-Fc chimeric-blocking reagent (Fig. 6D).

## DISCUSSION

These results demonstrate the existence of widely distributed langerin^+^ DCs that have variable expression of CD1a^+^ and are closely related to CD1c^+^ DCs. In the recently proposed classification of mammalian DCs, this subset is related to CD11b^+^ DCs of mice and is known as DC2 [2]. The expression of langerin is low but distinct and is found in sites that do not normally contain LCs, including lymphoid and nonlymphoid tissues. Langerin is not expressed by the CD141^high^ XCR1^+^ cross-presenting DC [3], and conversely, langerin^+^ CD1c^+^ DCs do not express the characteristic markers of cross-presenting DCs. The absence of langerin from human DC1 is an important species difference between mice and humans, contrasting with the consistent expression of langerin by mouse CD8^+^/CD103^+^ DC1 [18–20]. It should be noted, however, that approximately one-third of langerin^+^ DCs in murine nonlymphoid tissues does not express the DC1 marker CD103, suggesting expression on additional DC populations that may include some DC2 [20].

In vivo experiments with HSCT recipients and patients with DC deficiency show that langerin^+^ DCs and CD1a^+^ dermal DCs have parallel homeostasis with blood monocytes and DCs and are not derived from LCs. Finally, freshly isolated blood CD1c^+^ DCs contain langerin mRNA and up-regulate langerin surface-antigen expression in response to serum or TGF-*β* within 18 h. Together, these results indicate that CD1c^+^ myeloid DCs can be induced to express langerin and that in vivo, a fraction of these cells is langerin^+^, possibly through the action of local environmental factors, such as TGF-*β*.

Langerin has not been detected on tissue DCs of the skin and lung by previous studies [7, 10], although langerin^+^ DCs were reported in human colon [11], and there is reference to trace levels of langerin expression in human blood [28, 29]. TGF-*β* is able to up-regulate expression by CD1c^+^ blood DCs, suggesting that langerin expression in tissues may be induced by epithelial or autocrine TGF-*β* [30–32]. The use of an ALK-3-blocking reagent implicates similar TGF-*β* signaling pathways to those involved in the induction of langerin on progenitor cell populations [24]. One caveat of our experiments is that in vitro preparation of skin by enzymatic digest may have enhanced the level of langerin expression. However, our results also show that langerin^+^ DCs are detectable in situ by immunofluorescence and also found in lung, liver, and LN, tissues that do not require long preparative manipulation.

Expression of langerin by CD34^+^ progenitors and CD14 monocytes is well described after prolonged in vitro culture [8, 21–24, 31–33]. These techniques are usually designed to make LCs, although they may also generate langerin^+^ DCs similar to the phenotype that we describe in vivo. Several investigators have demonstrated Birbeck granules in in vitro-derived LCs. We were unable to find these organelles in primary langerin^+^ DCs. This may have been hampered by effacement of ultrastructural features following cell sorting, although it is notable that langerin^+^ DCs in mice also express less langerin than LCs and to our knowledge, have not been shown to contain Birbeck granules [15–17].

The finding that CD1c^+^ DCs can express langerin may have relevance to the pathogenesis of LCH, a rare, low-grade myeloproliferative-inflammatory disorder characterized by an abnormal accumulation of langerin^+^ CD1a^+^ myeloid cells. Comparison of a limited number of surface antigens confirmed that LCH cells are quite different to LCs [32, 34–37]. The lack of EpCAM, lower CD1a, and expression of CD11b, CD13, and CD33 by LCH cells is also a feature more in keeping with langerin^+^ CD1c^+^ DCs than LCs. A langerin^+^ DC origin for LCH is perhaps more consistent with the widespread distribution of multisystem LCH and absence of Birbeck granules in the liver and gastrointestinal tract [38]. Genetic tracking of mutations causing LCH have identified CD11c^+^ myeloid cells, which include CD1c^+^ DCs as potential precursors [39]. Further studies will be required to evaluate the potential links between CD1c^+^ myeloid DCs and this disease.

In summary, these results show that langerin expression is not confined to human LCs in vivo but is expressed at low level by a fraction of nonlymphoid and lymphoid CD1c^+^ DCs and is induced rapidly on blood CD1c^+^ DCs in vitro.

## AUTHORSHIP

V.B. designed and performed experiments, analyzed data, and wrote the paper. N.M., P.M., and R.D. performed experiments and analyzed data. S.P. and S.C. performed experiments. M.H. designed and performed experiments and analyzed data. M.C. designed experiments, analyzed data, and wrote the paper.

## Abbreviations

ALK-3: activin receptor-like kinase 3
APC: allophycocyanin
BMP-7: bone morphogenetic protein 7
CLEC9: C-type lectin domain family 9
DC: dendritic cell
EpCAM: epithelial cell adhesion molecule
FISH: fluorescence in situ hybridization
HSCT: hematopoietic stem cell transplant
LC: Langerhans cell
LCH: Langerhans cell histiocytosis
LN: lymph node
NECL2: nectin-like molecule 2
qPCR: quantitative PCR
XCR1: chemokine (C motif) receptor 1
